# Enabling deep-space experimentations on cyanobacteria by monitoring cell division resumption in dried *Chroococcidiopsis* sp. 029 with accumulated DNA damage

**DOI:** 10.3389/fmicb.2023.1150224

**Published:** 2023-05-17

**Authors:** Claudia Fagliarone, Claudia Mosca, Giorgia Di Stefano, Stefan Leuko, Ralf Moeller, Elke Rabbow, Petra Rettberg, Daniela Billi

**Affiliations:** ^1^Department of Biology, University of Rome Tor Vergata, Rome, Italy; ^2^PhD Program in Cellular and Molecular Biology, Department of Biology, University of Rome Tor Vergata, Rome, Italy; ^3^Aerospace Microbiology Research Group, Radiation Biology Department, Institute of Aerospace Medicine, German Aerospace Center (DLR), Cologne, Germany; ^4^Department of Natural Sciences, University of Applied Sciences Bonn-Rhein-Sieg (BRSU), Rheinbach, Germany; ^5^Astrobiology Research Group, Radiation Biology Department, Institute of Aerospace Medicine, German Aerospace Center (DLR), Cologne, Germany

**Keywords:** outer space, DNA damage, cell division, Fe-ion radiation, desert cyanobacteria

## Abstract

Cyanobacteria are gaining considerable interest as a method of supporting the long-term presence of humans on the Moon and settlements on Mars due to their ability to produce oxygen and their potential as bio-factories for space biotechnology/synthetic biology and other applications. Since many unknowns remain in our knowledge to bridge the gap and move cyanobacterial bioprocesses from Earth to space, we investigated cell division resumption on the rehydration of dried *Chroococcidiopsis* sp. CCMEE 029 accumulated DNA damage while exposed to space vacuum, Mars-like conditions, and Fe-ion radiation. Upon rehydration, the monitoring of the *ftsZ* gene showed that cell division was arrested until DNA damage was repaired, which took 48 h under laboratory conditions. During the recovery, a progressive DNA repair lasting 48 h of rehydration was revealed by PCR-stop assay. This was followed by overexpression of the ftsZ gene, ranging from 7.5- to 9-fold compared to the non-hydrated samples. Knowing the time required for DNA repair and cell division resumption is mandatory for deep-space experiments that are designed to unravel the effects of reduced/microgravity on this process. It is also necessary to meet mission requirements for dried-sample implementation and real-time monitoring upon recovery. Future experiments as part of the lunar exploration mission Artemis and the lunar gateway station will undoubtedly help to move cyanobacterial bioprocesses beyond low Earth orbit. From an astrobiological perspective, these experiments will further our understanding of microbial responses to deep-space conditions.

## Introduction

Long-term human presence on the Moon and future settlements on Mars will depend on the development of self-sufficient life support systems that will minimize dependence on resupply from Earth. Microorganisms offer great promise to support deep-space exploration: A so-called biomanufactory was proposed for human outposts on Mars, based on *in situ* resource utilization and biologically driven subunits for needed bioprocesses (Berliner et al., 2021). In such a scenario, cyanobacteria are gaining considerable interest due to their ability to produce oxygen and their potential as bio-factories for space biotechnology/synthetic biology applications (for a review see Mapstone et al., 2022). The “PowerCell” concept proposed cultivating cyanobacteria with raw materials available on the Moon and Mars to feed the bacteria used in biotechnology/synthetic biology (Rothschild, 2016). Recently, it was shown that the biomass produced by the desert cyanobacterium *Chroococcidiopsis* sp. 029 can be used as feedstock for bacteria (Billi et al., 2021).

Future lunar exploration mission Artemis and the lunar Gateway station (Crusan et al., 2019; Smith et al., 2020) will undoubtedly offer a unique opportunity to advance the use of cyanobacterial bioprocesses in space. Microorganisms on board the International Space Station (ISS) have exhibited global alterations in metabolic functions and gene expression, causing variations in biofilm formation, sporulation, and virulence (for a review see Milojevic and Weckwerth, 2020). Many unknowns remain in bridging the gaps in knowledge required to move cyanobacterial bioprocesses from Earth to space, and, for realistic microbial bioprocesses in space, a deeper understanding of the recovery process after traveling in space and subsequent reactivation is mandatory. In NASA's BioSentinel CubeSat, launched as a secondary payload on Artemis-1, dried cells of *Saccharomyces cerevisiae* are scheduled to be reactivated during an 18-month permanence in deep space, meaning that DNA damage repair will be monitored in deep space (Massaro Tieze et al., 2020; Santa Maria et al., 2020).

The investigations performed using ground-based simulations or facilities installed outside the ISS offer a baseline testbed for deep-space experiments by providing insights into microbial survivability after they have traveled through space and undergone subsequent reactivation. Post-flight analyses of the rehydration of the cyanobacterium *Chroococcidiopsis* sp. 029 exposed to space and Mars-like conditions during the BOSS (Biofilms Organisms Surfing Space) space mission showed: i) the upregulation of DNA repair genes in cells exposed to space vacuum (Mosca et al., 2021) and ii) the absence of increased genome variants in cells exposed to Mars-like conditions (Napoli et al., 2022). An upregulation of DNA repair genes was also reported during the rehydration of dried cells irradiated with Fe ions (Mosca et al., 2022) during the STARLIFE irradiation campaign (Moeller et al., 2017).

These results on the recovery of dried *Chroococcidiopsis* sp. 029 with accumulated DNA damage laid the foundation for its suitability for deep-space experimentation, which is necessary to unravel the effect of reduced/microgravity on the recovery process. However, even though the role of DNA repair genes was reported, it is fundamental to assess the timeframe needed for DNA repair and cell division resumption upon rehydration. This knowledge might be vital to meet mission requirements for dried-sample implementation and real-time monitoring upon recovery, in case of extensive pre-flight periods and unguaranteed sample return.

The link between DNA damage repair and cell division arrest is provided by the regulation of the *ftsZ* gene expression. In bacteria such as *Escherichia coli*, when RecA binds to single-stranded DNA, it stimulates the autocatalytic cleavage of LexA. This is then followed by the expression of the SOS repair genes, including the *sulA* gene that causes cell division delay through the inhibition of the FtsZ polymerization (Kreuzer, 2013). A comparative genome analysis suggested that the *E. coli*-like SOS model might not be valid for all cyanobacteria: The *lexA* gene, which regulates the SOS system, is unevenly distributed among the analyzed genomes, while the *sulA* gene, which stalls cell division until DNA reparation, is ubiquitous (Cassier-Chauvat et al., 2016). Thus far, it is unknown whether the *E. coli*-like SOS model is present in *Chroococcidiopsis* sp. 029, although it was reported that FtsZ is a critical component of its cell division machinery (Billi, 2009).

The present study aimed to determine the timeframe between DNA damage repair and cell division resumption upon the rehydration of dried *Chroococcidiopsis* sp. 029 that has undergone DNA damage. Lesions were accumulated during the exposure to space and Mars-like conditions during the BOSS space experiment and Fe-ion radiation during the STARLIFE project.

The presence of genome lesions and their repair upon rehydration was determined by using a PCR-stop assay by amplifying a 4-kbp genome fragment. Cell division resumption was determined by monitoring the expression of the cell division *ftsZ* gene by quantitative polymerase chain reaction (RT-qPCR) during 48 h of rehydration.

## Materials and methods

### Cyanobacterial strain and sample preparation

*Chroococcidiopsis* sp. 029 was isolated by Roseli Ocampo-Friedmann from cryptoendolithic growth in sandstone in the Negev Desert (Israel) and maintained at the University of Rome Tor Vergata, as part of the Culture Collection of Microorganisms from Extreme Environments established by E. Imre Friedmann. Cultures were grown under routine conditions at 25°C in BG-11 liquid medium (Rippka et al., 1979) under a photon flux density of 40 μmol/m^2^/s provided by fluorescent cool-white bulbs. Dried samples for the BOSS space experiment of the EXPOSE-R2 space mission were prepared as previously reported (Billi et al., 2019). Biofilms were obtained by growing cyanobacterial cells on top of BG-11 agarized medium for about 2 months, following which they were allowed to air-dry for about 15 days under routine growth conditions and finally stored in the dark under laboratory conditions. Dried cells for the STARLIFE experiment were prepared as previously reported (Verseux et al., 2017). Briefly, cells from cultures in the early stationary phase were immobilized on Millipore filters, air-dried overnight in a sterile hood, and stored in the dark under laboratory conditions.

### Exposure to space vacuum and Mars-like conditions in low Earth orbit

**Figure 1:**
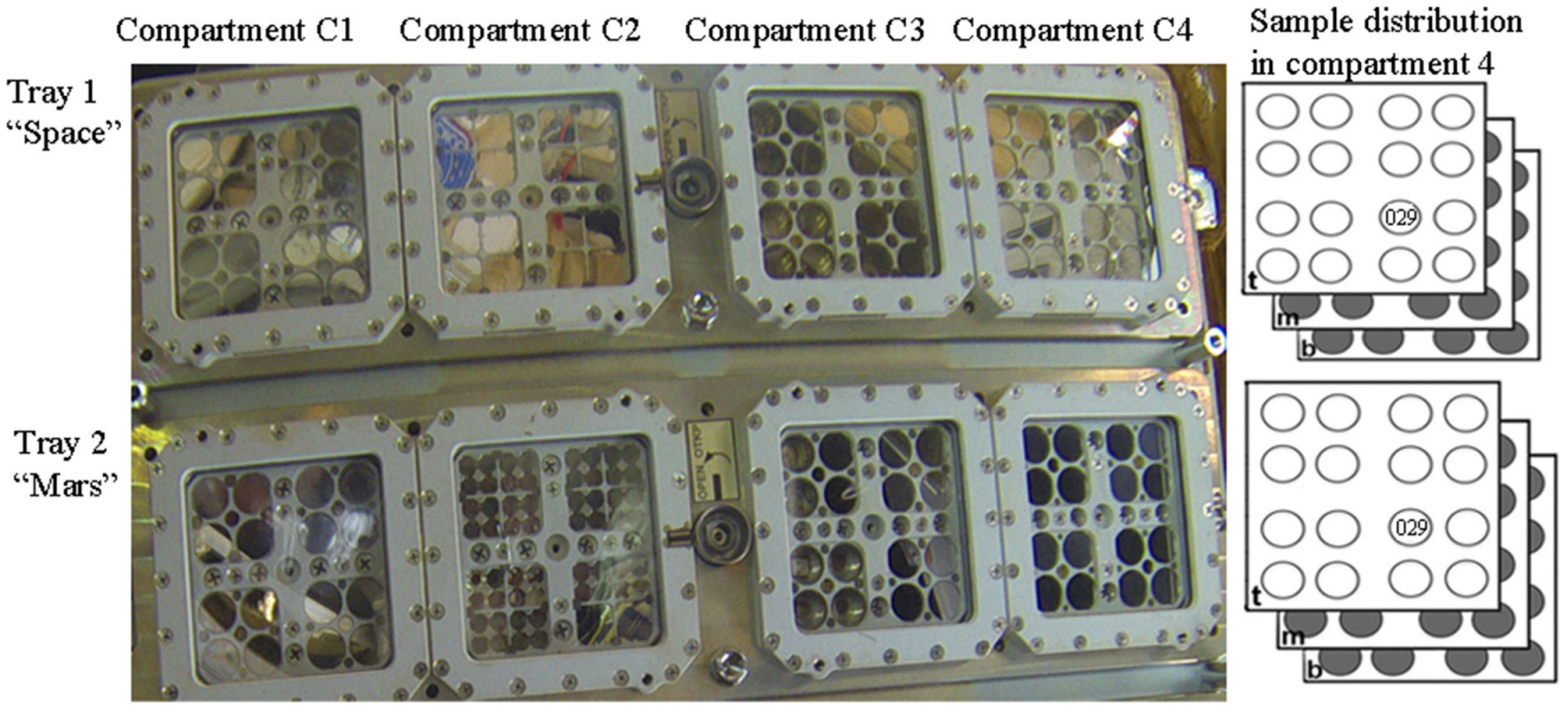
Sample distribution in the EXPOSE-R2 hardware. In compartment 4 of Tray 1 “Space” and Try 2 “Mars”, samples were allocated in top-layer (t), middle (m), and bottom-layer carrier (b). Dried biofilms of *Chroococcidiopsis* sp. 029 used in this study were exposed in the position 11 middle-layer of the carrier. Photo credits: Roscosmos and ESA.

In the context of the BOSS space experiment, which aimed to investigate biofilm resistance under space and Mars-like conditions in LEO (Cottin and Rettberg, 2019), dried biofilms of *Chroococcidiopsis* sp. 029 were accommodated in the compartment C4 of the tray 1 “space” and of the tray 2 “Mars” of the EXPOSE-R2 hardware (Figure 1). In the present study, we analyzed samples from the middle-layer carrier that were exposed for 672 days to a space vacuum and 0.5 Gy of cosmic ionizing radiation and temperature values ranging from −20.9°C to 57.98°C (Dachev et al., 2017; Rabbow et al., 2017). After retrieval to Earth, 2.5-year-old samples (considering 900 days from launch to space and sample shipping to the laboratory) were stored for an additional 4.5 years in an air-dried state under laboratory conditions. Control biofilms were air-dried and stored in the dark at room temperature for 7 years.

### Exposure to Fe-ion irradiation

Cells were first immobilized on Millipore filters and shipped to the Heavy Ion Medical Accelerator in Chiba (HIMAC, Gunma University Heavy Ion Medical Center, Japan) for irradiation, which was performed in June 2018 during the STARLIFE project, as previously reported (Mosca et al., 2022). After irradiation with 2 kGy of Fe ions, the air dried samples were stored in the dark at room temperature for 2 years.

### Sample recovery

Aliquots of dried cells and dried-exposed cells (about 25 mm^2^) were resuspended in 1 ml of BG-11 and allowed to recover under standard growth conditions as reported above.

### DNA damage evaluation

Genomic DNA was extracted from dried samples and from samples at different time points of rehydration by modifying a method previously reported (Billi et al., 1998). Briefly, the lysis of *Chroococcidiopsis* cells was achieved by adding hot phenol and glass beads. Then the pooled aqueous phases were first extracted with phenol–chloroform–isoamyl alcohol and followed by chloroform–isoamyl alcohol as described (Mosca et al., 2019). The PCR-stop assay was performed by amplifying a 4-kbp genome fragment using 6 ng of genomic DNA in 12-μl reaction mixtures containing 0.5 μM each of primers Chroo-4 K-2-F (5′-GCTACTCGTTGCTTTGCGTC-3′) and Chroo-4 K-2-R (5′-TTCCCCATACTTTGCTTCCCA-3′) and 6 μl of High-Fidelity Master Mix (Thermo Fisher Scientific, Waltham, MA, USA) as described (Mosca et al., 2019). A no-template control was included. Each one of the 12-μl PCR reaction mixtures was loaded onto a 1.5% agarose gel containing 0.5 mg/ml of ethidium bromide, subjected to electrophoresis for about 1 h at 90 V, and visualized with a trans-illuminator.

### Gene expression upon recovery

At different time points of rehydration, total RNA was extracted as described (Fagliarone et al., 2020). Real-time reactions were performed in 12 μl, including 1 μg of cDNA template, 6 μl of iTaq^TM^ universal SYBR^®^ Green Supermix (Bio-Rad Laboratories, Hercules, CA, USA), and 400 nM of forward primer chrftsZ-F (5′-GTTGACTTTGCCGATGTCCG-3′) and reverse primer chrftsZ-R (5′-CTTCTCTGGCACGGGACTTT-3′) that were designed based on a 473-bp fragment previously identified (Billi, 2009). PCR cycling conditions were as follows: a cycle of 95°C for 10 min, then 40 cycles of 95°C for 15 s, and 60°C for 1 min, followed by a ramp from 60 to 95°C for the melting curve stage. Quantification was performed using the Ct comparative method (2^−Δ*ΔCt*^ method) and using the 16S rRNA gene as the housekeeping gene (GenBank accession number AF279107) and employing the primers chr16S-F (5′-TACTACAATGCTACGGACAA-3′) and chr16S-R (5′-CCTGCAATCTGAACTGAG-3′). All cDNA quantities were normalized to 16S rRNA quantities, and fold changes of gene transcripts were compared to the values of non-rehydrated samples set as 1. Values >1 were considered upregulated, and values <1 were considered downregulated. Five rehydration time points (1, 6, 12, 24, and 48 h) were evaluated for dried cells exposed to Fe ions and 7 years of dried storage. Three rehydration time points (1, 6, and 48 h) were tested for dried biofilms exposed during the BOSS space mission, due to the limited availability of samples. In each PCR protocol, three replicates were performed, and no-template control was included.

### Statistical analysis

Only one sample for each condition was allocated in the EXPOSE-R2 facility. Therefore, three replicates for biologically distinct samples (different cultures) exposed to air-drying under laboratory conditions and Fe-ion irradiation were used, as well as three replicates of a single sample exposed to space vacuum and Mars-like conditions. Data are shown with standard deviation, and significance was assessed by using Student's *t*-test.

## Results

### DNA damage accumulated in dried cells and its recovery upon rehydration

**Figure 2:**
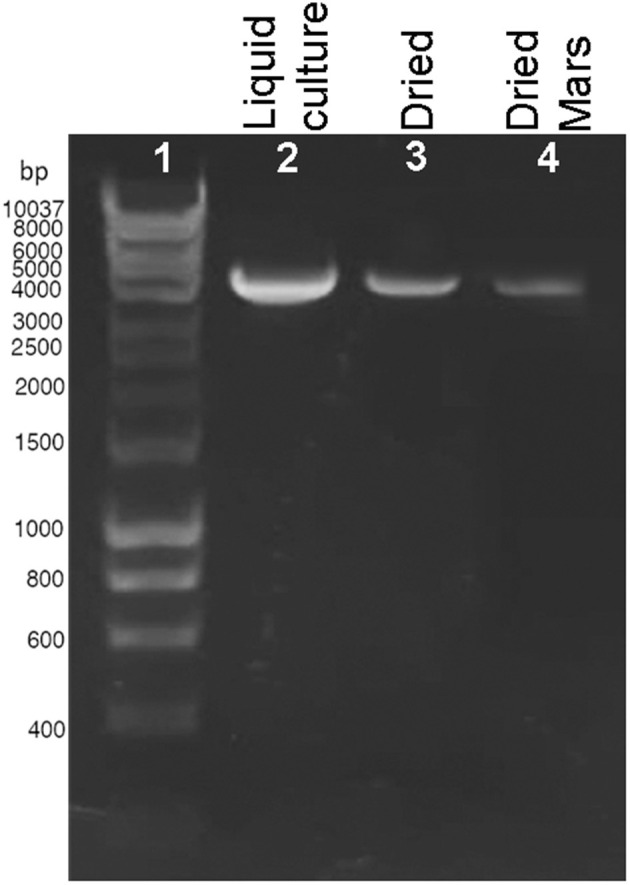
DNA damage accumulation in *Chroococcidiopsis* sp. 029 evaluated by PCR-stop assay with a 4-kbp amplification target. Control liquid culture (lane 2), dried biofilms maintained for 7 years in the dark, at room temperature (lane 3), and dried biofilms exposed to Mars-like conditions (lane 4). Lane 1: Hyperladder 1 kbp (Bioline Meridian Life Science, Memphis, TN, USA).

The presence of DNA damage was qualitatively determined by using a PCR-stop assay that evaluates the genome suitability as a template for PCR amplifications since lesions impede DNA polymerase progression (Kumar et al., 2004). When a 4-kbp genome fragment was amplified as a target, a reduction of the amplicon intensity was observed in dried biofilms exposed to Mars-like conditions (Figure 2, lane 4) compared to control liquid culture (Figure 2, lane 2) and dried control biofilms maintained in the dark at room temperature for 7 years (Figure 2, lane 3). No amplicons occurred in the PCR-stop assay performed with no genomic DNA (not shown). DNA damage in dried cells exposed to a space vacuum and to 2 kGy of Fe ions was previously reported (Mosca et al., 2021, 2022).

**Figure 3:**
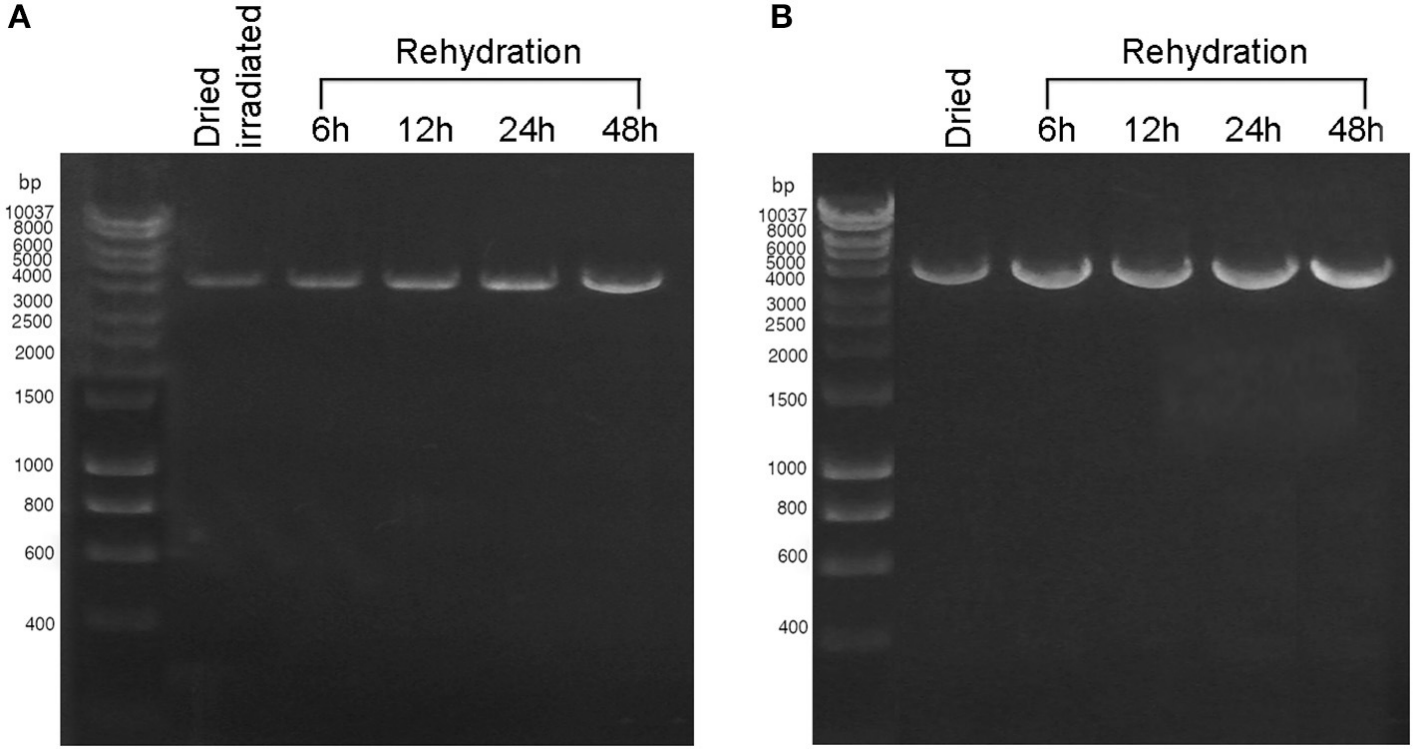
DNA damage restoration upon the rehydration of *Chroococcidiopsis* sp. 029 evaluated by PCR-stop assay with a 4-kbp amplification target. **(A)** Dried cells were exposed to 2 kGy of Fe ions (lane 2) and after 6, 12, 24, and 48-h recovery (lanes 3–6). **(B)** Dried cells were stored for 7 years in the dark at room temperature (lane 2) and after 6, 12, 24, and 48 h of recovery (lanes 3–6). Lane 1: Hyperladder 1 kbp (Bioline Meridian Life Science, Memphis, TN, USA).

During 48 h of rehydration, the 4-kbp amplicon was gradually restored in both dried cells exposed to 2 kGy of Fe ions (Figure 3A) and dried biofilms maintained in the dark at room temperature for 7 years (Figure 3B). For both samples, the intensity of the amplicon band continued to increase after 6 h of rehydration (Figures 3A, B), and no amplicons were observed in the PCR-stop assay performed with no genomic DNA (not shown). Due to the limited sample availability of the dried samples employed in the BOSS space mission, the DNA recovery during rehydration was not evaluated.

### *ftsZ* gene expression upon the rehydration of dried cells with damaged DNA

**Figure 4:**
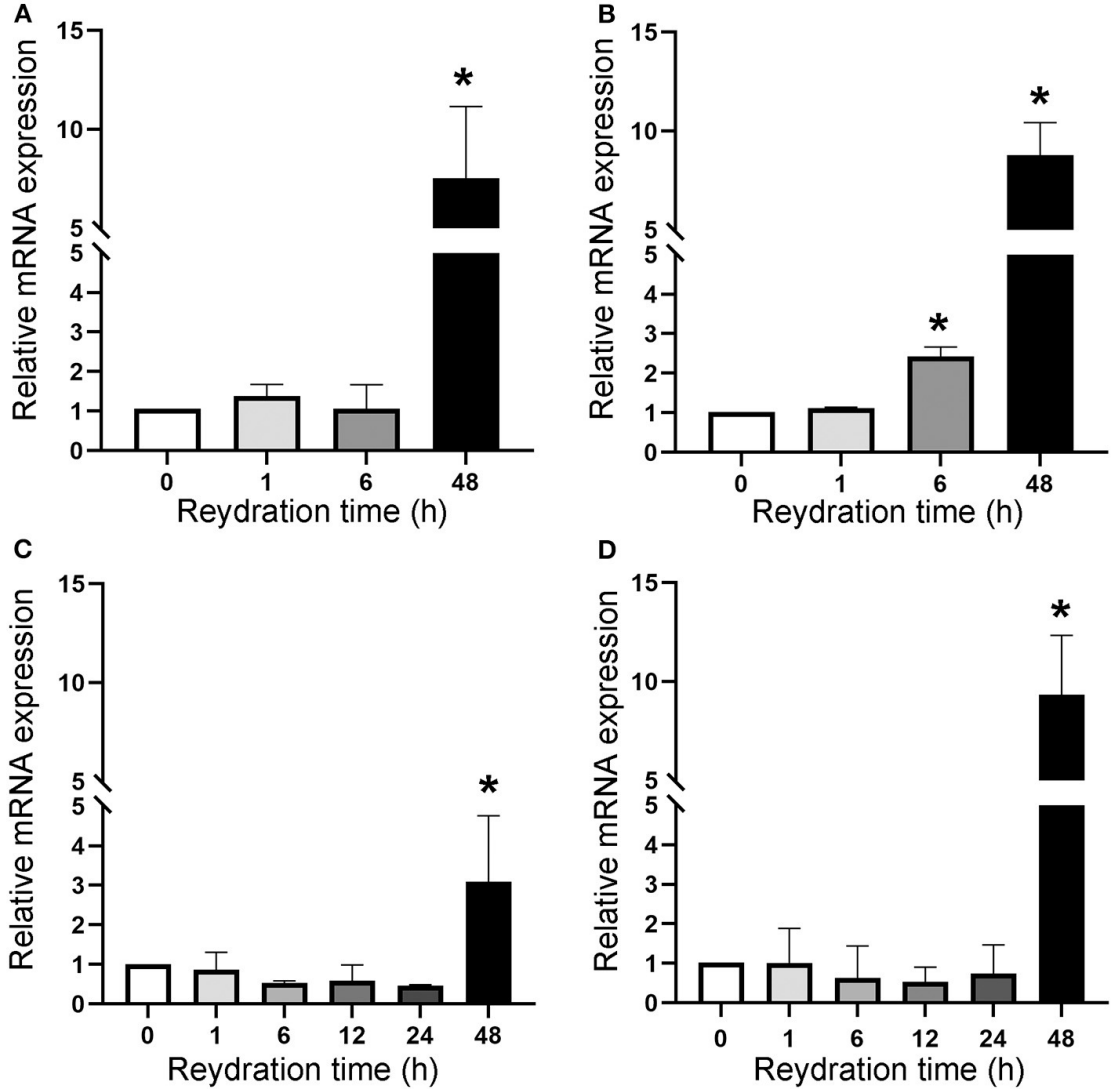
Expression of *ftsZ* gene upon the recovery of dried biofilms of *Chroococcidiopsis* sp. 029 exposed to space vacuum **(A)**, Mars-like conditions **(B)**, irradiation with Fe ions **(C)**, and in dried biofilms maintained for 7 years in the dark at room temperature **(D)**. Values from dried samples were considered control and set as 1. Data represent mean ± SD (*n* = 3), *p < 0.05.

The cell division resumption was monitored during the 48 h of rehydration of dried cells with accumulated DNA damage, by determining the expression of the *ftsZ* gene (Figure 4). Dried biofilms exposed to a space vacuum did not show any upregulation of the *ftsZ* gene during 1 h and 6 h of rehydration, while this gene was 7.5-fold upregulated after 48 h of rehydration, compared to the non-rehydrated sample (Figure 4A). In comparison to the non-rehydrated sample, dried biofilms exposed to Mars-like conditions showed a 2.5-fold increase in the transcriptional level of the ftsZ gene after 6 h and a 9-fold increase after 48 h recovery (Figure 4B). During the rehydration of dried cells irradiated with 2 kGy of Fe ions, the *ftsZ* gene showed a 3-fold upregulation after 48 h of rehydration (Figure 4C). The *ftsZ* gene was not upregulated during 1, 6, 12, and 24 h of rehydration in biofilms that had been air-dried in the dark for 7 years at room temperature. However, the gene was 10.4-fold over-expressed after 48 h of rehydration compared to the non-rehydrated sample (Figure 4D).

## Discussion

With the upcoming long-term space explorations, it is becoming increasingly important to broaden our understanding of microbial bioprocesses toward moving the same from Earth to space. Cyanobacteria are expected to play a key role in supporting long-term human presence on the Moon and future settlements on Mars (Mapstone et al., 2022). Thus, a deeper understanding of their recovery process after traveling in space and subsequent reactivation is very relevant.

Here, the timeframe required for DNA repair and cell division resumption under laboratory growth conditions was assessed during the rehydration of dried cells of the cyanobacterium *Chroococcidiopsis* sp. 029 that accumulated DNA damage during the BOSS space experiment (Mosca et al., 2021) and Fe-ion during the STARLIFE irradiation campaign (Mosca et al., 2022). Upon rehydration, the expression of the cell division gene *ftsZ* gene and the progression of the DNA damage restoration indicated that the cell division resumption required 48 h from the rehydration onset. Although the key role of FtsZ in the cell division machinery of *Chroococcidiopsis* sp. 029 was previously reported (Billi, 2009), the molecular mechanism involved in delaying cell division until DNA damage is repaired remains to be elucidated. However, a possible involvement of the *sulA* gene is anticipated since, in agreement with its ubiquity in cyanobacteria (Cassier-Chauvat et al., 2016), it is present in the genome of *Chroococcidiopsis* sp. 029 (Billi D. personal communication).

Due to the limited sample availability of the flight samples, the recovery of DNA damage was monitored by PCR-stop assay during the rehydration of air-dried cells and dried cells exposed to Fe-ion radiation, but not of dried cells exposed to Mars-like conditions and space vacuum. Although the PCR-stop assay did not determine the lesion type, the presence of DNA lesions in dried cells exposed to space vacuum and Fe-ion irradiation is known to be largely represented by single- and double-strand breaks and oxidative damage. Previous research has shown that genes of the homologous recombination RecF pathway and base excision are upregulated during the rehydration of dried cells exposed to space vacuum and Fe-ion irradiation (Mosca et al., 2021, 2022).

In the BOSS space mission, dried *Chroococcidiopsis* sp. 029 allocated in the middle-layer carrier of tray 1 “Space” was exposed to a space vacuum combined with 0.5 Gy of cosmic ionizing radiation (Dachev et al., 2017). Although this is a non-lethal dose for a cyanobacterium that can tolerate up to 12 kGy in the dried state (Verseux et al., 2017), it simulates the dose accumulated during a trip to Mars. Hence, it is anticipated that a 48-h recovery should be allowed in future space experiments aimed to investigate the effects of reduced gravity/microgravity on the DNA repair process upon the rehydration of dried cells that accumulated DNA damage.

In the BOSS space mission, dried *Chroococcidiopsis* sp. 029 allocated in the middle-layer carrier of tray 2 “Mars” experienced about 0.5 Gy of cosmic ionizing radiation in a Mars-like atmosphere (Rabbow et al., 2017). The exposure to 980 Pa of a gas mixture composed mainly of CO_2_ might explain reduced DNA damage as suggested by a 2.5-fold expression of the *ftsZ* gene after 6 h of rehydration, whereas a 9-fold expression occurred after 48 h of rehydration. The absence of oxygen in the tray 2 “Mars” might have caused reduced DNA damage as previously reported (Schulte-Frohlinde et al., 1994). In the context of microbial-based technologies for space settlements on Mars, a scenario of accidental failures causing exposure to a combination of low-pressure and CO2 should be taken into account (Cockell, 2022). Thus, this result is relevant for biomanufactory on Mars.

The suitability of *Chroococcidiopsis* sp. 029 for deep-space investigations was further supported by DNA repair and cell division resumption of dried cells exposed to Fe ions, which are the more lethal components of the galactic cosmic radiation since they induce complex DNA damage (Horneck et al., 2010). The DNA repair and cell division resumption of dried biofilms stored for 7 years in air-dried state in the dark at room temperature further highlighted the suitability of this cyanobacterium for space missions that require extensive pre-flight periods.

In conclusion, the monitoring of the *ftsZ* gene expression determined the timeframe for DNA repair and cell division resumption. Data from this study have important implications for further studies on the effects of reduced gravity/microgravity on this process, as well as, for deep-space missions requiring the implementation of dried samples and real-time monitoring upon reactivation. Within the upcoming lunar exploration mission Artemis and lunar Gateway station, deep-space experiments will facilitate further understanding toward moving microbial bioprocesses beyond Earth. From an astrobiological perspective, such experimentation will further our understanding of microbial response to deep-space conditions.

## Data availability statement

The original contributions presented in the study are included in the article, further inquiries can be directed to the corresponding author.

## Author contributions

DB: supervision, conceptualization, and writing—review and editing. PR: conceived and coordinated the BOSS experiment. RM: conceived and coordinated the STARLIFE experiment. ER and SL: contributed to sample exposure. CF, CM, and GDS: contributed to methodology, validation, and data analysis. All authors discussed the results and commented on the manuscript. All authors contributed to the article and approved the submitted version.
